# Is COVID-19 vaccine inequality undermining the recovery from the COVID-19 pandemic?

**DOI:** 10.7189/jogh.12.05020

**Published:** 2022-05-23

**Authors:** Ana Suárez-Álvarez, Ana J López-Menéndez

**Affiliations:** Department of Applied Economics, Faculty of Economics and Business, University of Oviedo, Spain

## Abstract

**Background:**

The devastating health and economic impact of the COVID-19 pandemic led to a global response in the development of effective vaccines to fight the disease in an extraordinarily short time. Both the development and the production of these vaccines opened a path of hope, but the inequality in vaccine distribution raises great concerns about the possibility of effectively eradicating the virus.

**Methods:**

It is particularly important to analyse the extent to which vaccines are equally distributed and investigate the possible effects of vaccine inequalities as well as its major drivers. For this purpose, this paper investigates the extent of equitable vaccine distribution using some well-known inequality measures and disentangles the main drivers of the share of vaccination. In addition, the paper analyses the relationship between the vaccination rate, the GDP growth, and the incidence of the coronavirus disease, with the aim of providing empirical evidence on existing relationships worldwide.

**Results:**

Our findings show that the situation is more challenging in less developed countries, especially African countries, due to weak health systems and low rates of vaccination. Moreover, we find a positive relationship between the share of vaccinated individuals and GDP. Consequently, the poorest, least developed countries with a lower rate of vaccine uptake will experience lower GDP growth.

**Conclusions:**

Vaccines and the vaccination process reveal the existing inequalities between countries and how they, in turn, impact the well-being of their citizens. People who live in less developed countries have a lower probability of being vaccinated, which translates into a greater probability of dying from COVID. Countries are seeing their economic future compromised by low vaccination levels, given the positive and significant relationship between the vaccination rate and GDP growth. In short, while some countries are trying to get back to some sort of normality, even with some pandemic protocols, the situation in less developed countries is more challenging due to weak health systems and low rates of vaccination. Consequently, the poorest, least developed countries with a lower rate of vaccine penetration will experience lower GDP growth, and the pandemic will have a greater effect on their economy due to low vaccination rates.

Since March 2020, the world has faced the COVID-19 pandemic, an unprecedented challenge with terrible health and socio-economic effects. In such a difficult context, the world has witnessed the rapid development of effective vaccines for fighting the disease. Both the development and the production of these vaccines opened a path of hope; however, vaccines are not reaching all parts of the world equally, which raises great concerns about the possibility of effectively eradicating the virus. According to the information collected by the WHO [1] and the United Nations [2], most vaccine doses have been distributed in high and upper-middle-income countries while other areas, especially African countries, are lagging in the vaccination process.

COVID-19 vaccine inequality will profoundly impact socio-economic recovery in low-income countries if urgent action to assure equitable access worldwide is not taken; it would also set back progress towards the Sustainable Development Goals (SDGs) [2,3]. Furthermore, unequal access to vaccines could increase other inequalities, since being unvaccinated generates economic and social disadvantages [4].

As the Director-General of the WHO said *“Vaccine inequity is the world’s biggest obstacle to ending this pandemic and recovering from covid-19. Economically, epidemiologically, and morally, it is in all countries' best interest to use the latest available data to make lifesaving vaccines available to all”.*

To make vaccines more accessible, in April 2020, several multilateral organisations have created the COVAX global initiative headed by the WHO, GAVI (the Vaccine Alliance), the Coalition for Epidemic Preparedness Innovations (CEPI) and United Nations International Children’s Emergency Fund (UNICEF) aimed at providing countries equal access to vaccines. Nevertheless, this initiative has not had the expected success so far and poor countries have had less access to vaccines than rich countries.

In this framework, it is particularly important to analyse the extent to which vaccines are equally distributed and investigate the possible effects of vaccine inequalities as well as their major drivers. This paper aims to demonstrate the extent of equitable vaccine distribution using some well-known inequality measures, disentangle the main drivers of vaccine distribution and analyse the relationship between the vaccination rate, the GDP growth, and the incidence of coronavirus disease. The overarching aim is to provide empirical evidence on the existing relationships globally.

Although several experts have expressed concerns about the distribution of vaccines and the vaccination process [4-8], studies analysing the current situation of both phenomena are limited. One study [9] analysed how to establish an ethical allocation for COVID-19 vaccines while another [10] examined vaccines distribution logistics. A study [11] analysed vaccine inequality and constructed a Lorenz curve [12] on the distribution of vaccines using data up to March 2021. Currently, there is a dearth of research focused on the current distribution of vaccines around the world, and consequently, a lack of in-depth analysis on the drivers of vaccination and its effects on economic growth.

The following research questions are considered: 1) To what extent are vaccines equally distributed, 2) what the reasons for the differences in vaccination between countries are and 3) how GDP growth, COVID incidence and vaccinations are related.

Our findings show that the share of fully vaccinated individuals has significantly increased during the period analysed and that there is a high level of inequality in vaccine distribution, with a downward trend. Despite the decrease in vaccination inequality, the fact that low vaccination rates persist in Africa is of great concern.

The regression analysis confirms these results, showing that less developed countries (with lower life expectancy and higher death rates due to cardiovascular diseases) have a lower proportion of fully vaccinated individuals, with vaccination shortages particularly significant in African countries.

Furthermore, the interconnections between the vaccination rate, the incidence of the coronavirus disease and the GDP growth have been explained through a simultaneous equations model, showing that the share of individuals fully vaccinated is positively correlated with the number of positive cases and confirming that vaccines positively affect economic growth.

The remainder of the paper has three main sections related to Methods, Results and Conclusions. We first present the data we use to carry out our studies and examine the distribution of vaccines around the world with the aim of analysing to what extent are vaccines equally distributed. Afterwards, we analyse the main drivers of the vaccination rates, to understand the reasons for the differences in vaccination between countries. We also explore the relationship between the vaccination rate and the incidence rate of COVID-19 and GDP growth using a simultaneous equation model. Finally, the Conclusions section briefly describes the main findings of the analyses carried out.

## METHODS

### At what extent are vaccines equally distributed?

Inequality in the distribution of vaccines is a matter of the utmost health and socio-economic interest. Since the effectiveness of vaccines is greatly affected by the number of doses received, we have decided to analyse the proportion of individuals vaccinated with a full schedule.

To properly analyse the level of inequality in COVID-19 vaccination, we need reliable data about each country’s vaccination process. We used the Our World in Data COVID-19 vaccination data set [13,14], a global public data set containing information about vaccinations for more than 150 countries.

This database provides daily data about the number of COVID-19 vaccinations administered in each country based on information collected from official sources: health ministries, government reports, and official social media accounts. In addition to the Our World in Data variables, we used information on population and GDP growth provided by IMF.

To track the evolution of the vaccination process for most countries, we use the values at the end of each month from May to December 2021. We started our analysis in May so we could gather a large sample of countries, since the vaccination process had already begun at that time in most countries. We used the share of fully vaccinated individuals in each country as the reference variable for measuring vaccine distribution.

### What are the reasons for the differences in vaccination between countries?

We will try to disentangle the reasons for the vaccination rate differences between countries. For this purpose, we analyse the major drivers of vaccination coverage by performing several regressions on the share of fully vaccinated people at the end of December 2021 to understand which variables are its main determinants.

### How are GDP growth and vaccinations related?

Inequitable vaccine distribution is not only an ethical problem: it is also worrisome from the health and economic points of view. An infectious disease like COVID-19 will remain a global threat as long as it exists anywhere; inequality in the vaccine distribution could allow new deadly variants to emerge and spread across the world, as explained by the United Nations [15]. Furthermore, vaccine inequality could have a negative impact on economic recovery, especially for low-income countries [15,16].

After analysing the determinants of the vaccination rate, we wanted to examine the relationship between the vaccination rate, the coronavirus disease incidence, and the GDP growth. Since these three concepts seem to be interconnected, we considered a Simultaneous Equation Model (SEM) as the best specification for understanding this relationship between variables as well as modelling both the socio-economic and health impact of the vaccine.

For this purpose, we specified a model including four equations with four endogenous variables; two of them were intended to capture the COVID incidence: the number of new COVID cases and deaths at the end of December 2021, smoothed per million. The two remaining equations considered the share of fully vaccinated individuals at the end of December and the GDP growth rate for 2021 (retrieved from the IMF) as dependent variables. The model was estimated using Three Stage Least Squares (3SLS).

## RESULTS

### At what extent are vaccines equally distributed?

**Figure 1:**
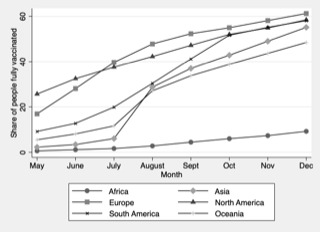
Evolution of the share of people fully vaccinated in each world region.

**Figure 1** shows the share of fully vaccinated individuals in each continent, which has significantly increased during the period analysed. At the end of December, the continent with the highest number of fully vaccinated individuals is Europe, followed by North America and South America.

Africa is the continent with the smallest share of fully vaccinated individuals, with less than 10% fully vaccinated individuals at the end of October. This fact reveals the unequal access countries have had to vaccines during this period and the moderate success of the COVAX initiative to make vaccines available for all countries. Additionally, many individuals in the African continent reported they would not accept a COVID-19 vaccine [17], which could exacerbate the differences observed in the share of fully vaccinated individuals.

**Figure 2:**
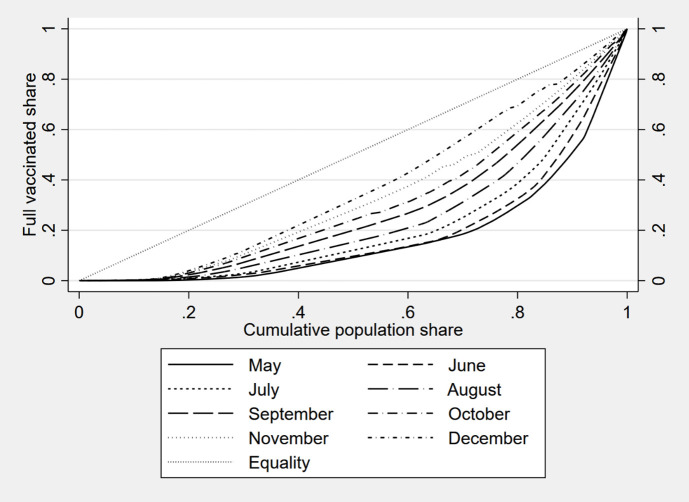
Lorenz curve of the distribution of vaccinated individuals by month.

To provide a more detailed picture of vaccine distribution in the world, **Table 1** shows some inequality indicators computed using around 140 countries. **Figure 2** shows the Lorenz curve of the vaccine´s distribution. The list of countries included in our analyses for each month can be found in the appendix (Table S1 in the **Online Supplementary Document**).

**Table 1:** Inequality indicators of the share of fully vaccinated individuals.

	May	June	July	August	September	October	November	December
Gini	0.63	0.58	0.55	0.48	0.43	0.39	0.36	0.33
GE(2)	0.80	0.60	0.50	0.36	0.28	0.23	0.20	0.17
p90/p50	6.74	4.88	4.03	2.59	2.20	1.87	1.76	1.63
p75/p25	32.40	22.59	15.16	8.68	5.16	4.70	3.74	2.84

We include several measures with the aim of having a comprehensive picture of vaccine inequality: the Gini index, the GE(2) and two percentile rations (p90/p50 and p75/p25). We use the Gini index as the most used income inequality measure with many desirable statistical properties, which allows an easy interpretation since it is a bounded measure [18].

Bearing in mind that a single inequality measure cannot capture all aspects of interest to researchers, we also decided to include a generalised entropy index in our study [19,20], and two well-known inequality ratios. For entropy measures, we consider the GE(2) index due to its sensitivity to what happened in the tails of the distribution whilst the Gini index is more sensitive to changes in the middle of the distribution. Finally, we complement the inequality analysis by incorporating two percentile ratios that allow us to make direct comparisons between the top and the centre of the distribution (p90/p50), and the central boundaries between which the central half of the observations are comprised (p75/p25).

The Lorenz curve represents the vaccine distribution among the population of the analysed countries. To construct the curve, countries are arranged in increasing order based on their cumulative share of fully vaccinated individuals. The horizontal axis of the graph includes the cumulative population share of countries whereas the vertical axis includes the cumulated share of fully vaccinated individuals. Therefore, a perfectly equal distribution of vaccines would be represented by a straight line of 45 degrees; the further the Lorenz curve is from this line, the more unequal the distribution will be.

Results from both the indicators in **Table 1** and the graph in **Figure 2** show the same trend regarding inequality in vaccination. Inequality in vaccine distribution measured by the share of fully vaccinated individuals in each country has decreased over the last months, meaning that vaccines reach more and more individuals in different countries.

These results agree with those obtained by [11], that also constructed a Lorenz curve for the distribution of vaccines but considering data up to March 31 of 2021. Although these authors found a higher level of inequality in the distribution of vaccines, their results also corroborate the observed downward trend.

Nevertheless, inequality is still high, especially bearing in mind that we measure inequality for the proportion of individuals with a completed vaccination schedule. Inequality measured by this variable is only expected to decrease. Consequently, the ideal scenario should be one in which everybody has received the vaccine. For the moment, we are still far from this desirable situation.

Moreover, it is important to notice that African countries have generally not increased the share of vaccinated individuals, meaning that the immunisation rate is almost steady overtime, as **Figure 1** illustrates. Based on these results, it is important to consider the reasons for the differences in vaccination rates between the different territories analysed.

### What are the reasons behind the differences in vaccination between countries?

Based on the share of fully vaccinated individuals, we have shown that inequalities between countries have decreased in recent months. However, inequality is still high considering that the ideal situation is one in which everyone has received the vaccine regardless of their country of residence.

**Table 2** shows the main results of the regressions performed using country-specific variables collected from Our World in Data, with the addition of a dummy variable which identifies African countries.

**Table 2:** Regressions on the share of fully vaccinated individuals at the end of December*

Variables/Regressions	(1)	(2)	(3)	(4)	(5)	(6)	(7)
**Population in 2020**	0.00191						
	(0.0101)						
**Share of vaccinated in May**	0.441‡	0.372‡	0.309‡	0.318‡	0.287†	0.266†	0.297‡
	(0.134)	(0.117)	(0.116)	(0.113)	(0.111)	(0.112)	(0.111)
**GDPpc ppp 2020**	0.000610§		0.000213†	0.000165	0.000154	0.000156	0.000215†
	(0.0000846)		(0.0000934)	(0.0000929)	(0.0000904)	(0.0000827)	(0.0000897)
**Africa**	-17.73§	-2.133	-1.890	3.050	-1.599	-9.676‡	-3.436
	(4.225)	(5.389)	(5.265)	(5.162)	(4.086)	(3.632)	(4.577)
**Asia**	5.925	9.460†	10.07†	14.24§	10.56§		8.130‡
	(3.620)	(4.397)	(4.269)	(3.832)	(2.951)		(3.068)
**North America**	2.476	2.768	2.333	6.481			-0.457
	(4.566)	(4.858)	(4.954)	(4.813)			(4.016)
**Oceania**	1.120	5.394	3.819	8.587			2.052
	(8.935)	(7.996)	(7.666)	(7.670)			(7.303)
**South America**	14.35‡	9.750	12.12†	16.05‡	12.01‡		10.52†
	(5.126)	(5.199)	(5.221)	(4.982)	(4.136)		(4.548)
**Aged 70 or older**		0.465					
		(0.529)					
**Cardiovascular death rate**		-0.0325‡	-0.0252†	-0.0310‡	-0.0345‡	-0.0306‡	-0.0272†
		(0.0117)	(0.0119)	(0.0118)	(0.0114)	(0.0105)	(0.0113)
**Diabetes prevalence**		-0.272	-0.248	-0.306	-0.124		
		(0.350)	(0.331)	(0.320)	(0.296)		
**Life expectancy**		2.125§	1.926§	1.472§	1.553§	1.850§	1.923§
		(0.331)	(0.324)	(0.351)	(0.347)	(0.285)	(0.278)
**Age 65 or older**			0.122				
			(0.364)				
**Median age**				0.709†	0.509		
				(0.293)	(0.262)		
**Constant**	29.38§	-109.9§	-100.00§	-88.07§	-84.34§	-86.41§	-98.66§
	(3.848)	(24.30)	(23.35)	(22.86)	(22.64)	(21.75)	(22.06)
**N**	146	142	141	141	141	143	143
**adj. R-sq**	0.686	0.775	0.792	0.800	0.800	0.780	0.793

*Standard errors in parentheses

†*P* < 0.05, ‡*P* < 0.01, §*P* < 0.001

From the regressions, we can see that countries with higher shares of vaccinated individuals in May 2021 are still the ones with a higher share of fully vaccinated individuals at the end of the year. Likewise, per capita GDP has had a moderately positive impact on the share of fully vaccinated individuals at the end of December 2021.

We also find that life expectancy has a positive impact on the proportion of vaccinated individuals, while the cardiovascular death rate has a significant negative impact. This means that, in countries where more individuals die from cardiovascular diseases, the percentage of vaccination is lower. Since these two variables could indicate the country’s degree of development, this suggests that less developed countries (with lower life expectancy and higher death rates for cardiovascular diseases) have a lower proportion of fully vaccinated individuals. Finally, regarding the African dummy, as expected, African countries have a significantly lower rate of individuals fully vaccinated, which is in line with our results.

### How are GDP growth and vaccination shares related?

**Table 3** shows the results of the Simultaneous Equation Model (SEM), which has been estimated using Three Stage Least Squares (3SLS).

**Table 3:** Multiple Equations Model

	Coefficient	Std. Error	Z	*P*-value
**Equation 1**	**Dependent variable:**	**Share of vaccinated**		
Constant	-190.10	23.81	-7.99	0.00‡
GDP Growth 2021	-13.40	3.69	-3.63	0.00‡
Life Expectancy	4.06	0.48	8.37	0.00‡
Meand dependent var	45.09	SD dependent var		26.60
Sum squared residuals	259750.90	SE or regression		42.77
R-squared	0.14	Adjusted R-squared		0.13
Equation 2	Dependent variable:	Covid		
	Coefficient	Std. Error	Z	*P*-value
Constant	386.97	222.35	1.74	0.08*
Share of vaccinated	11.36	2.75	4.13	0.00‡
Cardiovascular deaths	-1.08	0.52	-2.06	0.04†
Diabetes prevalence	-31.90	14.31	-2.23	0.03†
Meand dependent var	389.28	SD dependent var		718.31
Sum squared residuals	56970188.00	SE or regression		633.40
R-squared	0.22	Adjusted R-squared		0.20
Observations: 142				
Equation 3	Dependent variable:	Covid Deaths		
	Coefficient	Std. Error	Z	*P*-value
Constant	-5.38	0.79	-6.78	0.00‡
Share of vaccinated	-0.09	0.02	-6.09	0.00‡
Median Age	0.36	0.04	9.08	0.00‡
Meand dependent var	1.70	SD dependent var		2.99
Sum squared residuals	840.04	SE or regression		2.43
R-squared	0.35	Adjusted R-squared		0.34
Observations: 142				
Equation 4	Dependent variable:	GDP Growth in 2021		
	Coefficient	Std. Error	Z	*P*-value
Constant	3.75	0.54	7.01	0.00‡
Share of vaccinated	0.03	0.01	2.52	0.01†
GDP	0.00	0.00	−2.384	0.02†
				
Meand dependent var	4.64	SD dependent var		3.09
Sum squared residuals	1310.62	SE or regression		3.04
R-squared	0.03	Adjusted R-squared		0.02
Observations: 142				

*P < 0.10, †*P* < 0.05, ‡*P* < 0.01

Because of the sample heterogeneity, the results of the estimated model show discrete levels of the coefficient of determination, especially low for the growth equation. Regarding the role of vaccinations (which is the objective of the paper), the results of the estimated models show that the countries with a higher level of development (approximated by life expectancy) in which economic growth has been hampered by the pandemic have a higher rate of vaccine uptake. This explains the negative sign of the GDP growth coefficient.

Moreover, the share of fully vaccinated individuals is positively correlated with the number of positive cases, which could be explained by three aspects: First, those countries that suffered the most from the COVID pandemic have made a great effort to reach a high rate of vaccinated individuals, especially in Europe. Second, the higher rate of testing in these areas may affect the number of positive cases. Third, vaccines are not sterilizing and therefore do not make the infections disappear, but they do reduce the severity of the disease. This last reasoning is corroborated by the results from the third equation of the model, which shows that vaccines significantly reduce deaths.

Finally, regarding the relationship between vaccines and economic growth, our model confirms that vaccines positively affect economic growth, which implies that countries that have been able to achieve a high rate of vaccination among their population will recover faster from the economic shock produced by the pandemic.

## CONCLUSIONS

Vaccines are playing an outstanding role to fight the COVID-19 disease globally. The empirical evidence shows a great level of inequality in vaccine distribution worldwide, although this inequality has decreased over the last months. The analyses carried out in this paper show that most developed countries have achieved a higher rate of vaccination among their population, while less developed countries, especially those of the African continent, have not been able to vaccinate their population effectively. These countries have started vaccination campaigns later than developed countries and they vaccinate the population at a much slower rate, so they are not able to reduce their distance from developed countries in terms of vaccinations.

Regarding the reasons for the differences in vaccination between countries, the degree of development of countries is crucial in explaining the proportion of vaccinated individuals. Using the variables of life expectancy and cardiovascular death rate as proxies for development, we can see that the variable former has a positive impact on the proportion of vaccinated individuals while the latter has a negative impact.

Moreover, with the simultaneous equation model specified above, we find that the share of individuals fully vaccinated has a positive effect on economic growth, which shows that vaccination is not only beneficial for individuals’ health but also for the economy.

Vaccines and the vaccination process reveal the existing inequalities between countries and how these, in turn, impact the well-being of their citizens. Thus, those people who live in less developed countries have a lower probability of being vaccinated, which translates into a greater probability of dying from COVID.

Likewise, given the positive and significant relationship between the vaccination rate and GDP growth, low vaccination levels compromise a country’s economic future. While some countries try to get back to some sort of normality, even with some pandemic protocols, the situation in less developed countries is more challenging, due to weak health systems and low rates of vaccination. Consequently, the poorest, least developed countries with a lower rate of vaccine penetration will experience lower GDP growth, and the pandemic will have a greater effect on their economy due to low vaccination rates.

To sum up, our paper makes some important contributions to the COVID-19 vaccination process. Our analyses shed some light on the current state of the vaccination process and disentangle the main drivers of the vaccination rate between countries. We also analyse the socio-economic and health impacts of the vaccine as well as the relationship between the vaccination rate, the incidence of the coronavirus disease and the GDP growth using a Simultaneous Equations Model.

In view of our findings, we believe COVAX should make bigger efforts to guarantee access to vaccines for everyone. In the same way, it would be advisable for the most developed countries with greater economic resources to help less developed countries to access vaccines. COVID is a global problem, and no one should be left out of the solution.

Our findings open new possibilities for further research. On the one hand, access to broader and more up-to-date information would allow us to expand the existing evidence and increase the strength of the results. On the other hand, it would be of great interest to conduct an in-depth analysis of the causes of the existing vaccination inequality, distinguishing between institutional factors (mainly related to distribution) and individual attitudes (as hesitance or lack of information) by implementing, for example, the Inequality of Opportunity approach [21,22]. All these contributions would be very useful in bringing us closer to the common goal of reducing existing inequality as much as possible.

## Additional material


Online Supplementary Document
