# Supplementary material for: Is COVID-19 vaccine inequality undermining the recovery from the COVID-19 pandemic?
Source: J Glob Health. 2022 May 23;12:05020. doi: 10.7189/jogh.12.05020 (PMC9126303; doi:10.7189/jogh.12.05020)
Supplement: Online Supplementary Document [file jogh-12-05020-s001.pdf]

Table A1. Availability of vaccination data by month

	May	June	July	August	September	October	November
Albania	X	X	X	X	X	X	X
Angola	X	X	X	X	X	X	X
Antigua and Barbuda	X	X	X	X	X	X	X
Argentina	X	X	X	X	X	X	X
Armenia	X	X	X	X	X	X	X
Australia	X	X	X	X	X	X	X
Austria	X	X	X	X	X	X	X
Azerbaijan	X	X	X	X	X	X	X
Bahrain	X	X	X	X	X	X	X
Bangladesh	X	X	X	X	X	X	X
Barbados	X	X	X	X	X	X	X
Belarus	X	X	X	X	X	X	X
Belgium	X	X	X	X	X	X	X
Belize	X	X	X	X	X	X	X
Benin	X	X	X	X	X	X	X
Bolivia	X	X	X	X	X	X	X
Bosnia and Herzegovina	X	X	X	X	X	X	
Botswana	X	X	X	X	X	X	X
Brazil	X	X	X	X	X	X	X
Brunei	X	X	X	X	X	X	X
Bulgaria	X	X	X	X	X	X	X
Burkina Faso	X	X	X	X	X	X	X
Cambodia	X	X	X	X	X	X	X
Cameroon	X	X	X	X	X	X	X
Canada	X	X	X	X	X	X	X
Cape Verde	X	X	X	X	X	X	X
Chad	X	X	X	X	X	X	X
Chile	X	X	X	X	X	X	X
Colombia	X	X	X	X	X	X	X
Costa Rica	X	X	X	X	X	X	X
Croatia	X	X	X	X	X	X	X
Cyprus	X	X	X	X	X	X	X
Czechia	X	X	X	X	X	X	X
Democratic Republic of Congo	X	X	X	X	X	X	X
Denmark	X	X	X	X	X	X	X
Djibouti	X		X	X	X	X	X
Dominica	X	X	X	X	X	X	X
Dominican Republic	X	X	X	X	X	X	X
Ecuador	X	X	X	X	X	X	X
Egypt	X	X	X	X	X	X	X
El Salvador	X	X	X	X	X	X	X
Equatorial Guinea	X	X	X	X	X	X	X
Estonia	X	X	X	X	X	X	X
Ethiopia	X	X	X	X	X	X	X

Finland	X	X	X	X	X	X	X
France	X	X	X	X	X	X	X
Gabon	X	X	X	X	X	X	X
Gambia		X	X	X	X	X	X
Georgia	X	X	X	X	X	X	X
Germany	X	X	X	X	X	X	X
Ghana	X	X	X	X	X	X	X
Greece	X	X	X	X	X	X	X
Guatemala	X	X	X	X	X	X	X
Guinea	X	X	X	X	X	X	X
Guinea-Bissau	X	X	X	X	X	X	X
Guyana	X	X	X	X	X	X	X
Haiti	X	X	X	X	X	X	X
Honduras	X	X	X	X	X	X	X
Hong Kong	X	X	X	X	X	X	X
Hungary	X	X	X	X	X	X	X
Iceland	X	X	X	X	X	X	X
India	X	X	X	X	X	X	X
Indonesia	X	X	X	X	X	X	X
Iran	X	X	X	X	X	X	X
Iraq	X	X	X	X	X	X	X
Ireland	X	X	X	X	X	X	X
Israel	X	X	X	X	X	X	X
Italy	X	X	X	X	X	X	X
Jamaica	X	X	X	X	X	X	X
Japan	X	X	X	X	X	X	X
Jordan	X	X	X	X	X	X	X
Kazakhstan	X	X	X	X	X	X	X
Kenya	X	X	X	X	X	X	X
Kosovo	X	X	X	X	X	X	X
Kyrgyzstan	X	X	X	X	X	X	X
Laos	X	X	X	X	X	X	X
Latvia	X	X	X	X	X	X	X
Lebanon	X	X	X	X	X	X	X
Liberia	X	X	X	X	X	X	X
Libya	X	X	X	X	X	X	X
Lithuania	X	X	X	X	X	X	X
Macao	X	X	X	X	X	X	X
Malawi	X	X	X	X	X	X	X
Malaysia	X	X	X	X	X	X	X
Maldives	X	X	X	X	X	X	X
Mali	X	X	X	X	X	X	X
Malta	X	X	X	X	X	X	X
Mauritania	X	X	X	X	X	X	X
Mauritius	X	X	X	X	X	X	X
Mexico	X	X	X	X	X	X	X

Moldova	X	X	X	X	X	X	X
Mongolia	X	X	X	X	X	X	X
Montenegro	X	X	X	X	X	X	X
Morocco	X	X	X	X	X	X	X
Mozambique	X	X	X	X	X	X	X
Namibia	X	X	X	X	X	X	X
Nepal	X	X	X	X	X	X	X
Netherlands	X	X	X	X	X	X	X
New Zealand	X	X	X	X	X	X	X
Niger	X	X	X	X	X	X	X
Nigeria	X	X	X	X	X	X	X
North Macedonia	X	X	X	X	X	X	X
Norway	X	X	X	X	X	X	X
Oman	X	X	X	X	X	X	X
Pakistan	X	X	X	X	X	X	X
Panama	X	X	X	X	X	X	X
Papua New Guinea	X		X	X	X	X	X
Paraguay	X	X	X	X	X	X	X
Peru	X	X	X	X	X	X	X
Philippines	X	X	X	X	X	X	X
Poland	X	X	X	X	X	X	X
Portugal	X	X	X	X	X	X	X
Qatar	X	X	X	X			
Romania	X	X	X	X	X	X	X
Russia	X	X	X	X	X	X	X
Rwanda	X	X	X	X	X	X	X
Sao Tome and Principe	X	X	X	X	X	X	X
Saudi Arabia		X	X	X	X	X	X
Senegal	X	X	X	X	X	X	X
Serbia	X	X	X	X	X	X	X
Sierra Leone	X	X		X	X	X	X
Singapore	X	X	X	X	X	X	X
Slovakia	X	X	X	X	X	X	X
Slovenia	X	X	X	X	X	X	X
Somalia	X	X	X	X	X	X	X
South Africa	X	X	X	X	X	X	X
South Korea	X	X	X	X	X	X	X
South Sudan	X	X	X	X	X	X	X
Spain	X	X	X	X	X	X	X
Sri Lanka	X	X	X	X	X	X	X
Sudan	X	X	X	X	X	X	X
Suriname	X	X	X	X	X	X	X
Sweden	X	X	X	X	X	X	X
Switzerland	X	X	X	X	X	X	X
Taiwan	X	X	X	X	X	X	X
Tajikistan	X	X	X	X	X	X	X

Thailand	X	X	X	X	X	X	X
Trinidad and Tobago	X	X	X	X	X	X	X
Tunisia	X	X	X	X	X	X	X
Turkey	X	X	X	X	X	X	X
Ukraine	X	X	X	X	X	X	X
United Arab Emirates		X	X	X	X	X	
United Kingdom	X	X	X	X	X	X	X
United States	X	X	X	X	X	X	X
Uruguay	X	X	X	X	X	X	X
Uzbekistan	X	X	X	X	X	X	
Venezuela	X	X	X	X	X	X	X
Vietnam	X	X	X	X	X	X	X
Zambia	X	X	X	X	X	X	X
Zimbabwe	X	X	X	X	X	X	X
Number of countries available	147	148	149	150	149	149	146

Table A2. Countries included in the Simultaneous Equations Model

Albania	Laos
Angola	Latvia
Antigua and Barbuda	Lebanon
Argentina	Liberia
Armenia	Lithuania
Australia	Malawi
Austria	Malaysia
Azerbaijan	Maldives
Bahrain	Mali
Bangladesh	Malta
Barbados	Mauritania
Belarus	Mauritius
Belgium	Mexico
Belize	Moldova
Benin	Mongolia
Bolivia	Montenegro
Botswana	Morocco
Brazil	Mozambique
Brunei	Namibia
Bulgaria	Nepal
Burkina Faso	Netherlands
Cambodia	New Zealand
Cameroon	Niger
Canada	Nigeria
Cape Verde	North Macedonia
Chad	Norway
Chile	Oman
Colombia	Pakistan
Costa Rica	Panama
Croatia	Papua New Guinea
Cyprus	Paraguay
Czechia	Peru
Democratic Republic of Congo	Philippines
Denmark	Poland
Djibouti	Portugal
Dominican Republic	Romania
Ecuador	Russia
Egypt	Rwanda
El Salvador	Sao Tome and Principe
Equatorial Guinea	Saudi Arabia
Estonia	Senegal
Ethiopia	Serbia
Finland	Sierra Leone
France	Singapore

Gabon	Slovakia
Gambia	Slovenia
Georgia	Somalia
Germany	South Africa
Ghana	South Korea
Greece	South Sudan
Guatemala	Spain
Guinea	Sri Lanka
Guinea-Bissau	Sudan
Guyana	Suriname
Haiti	Sweden
Honduras	Switzerland
Hungary	Tajikistan
Iceland	Thailand
India	Trinidad and Tobago
Indonesia	Tunisia
Iran	Turkey
Iraq	Ukraine
Ireland	United Arab Emirates
Israel	United Kingdom
Italy	United States
Jamaica	Uruguay
Japan	Uzbekistan
Jordan	Venezuela
Kazakhstan	Vietnam
Kenya	Zambia
Kyrgyzstan	Zimbabwe
